# Supplementary material for: Sensitizing Staphylococcus aureus to antibacterial agents by decoding and blocking the lipid flippase MprF
Source: eLife. 2022 Jan 19;11:e66376. doi: 10.7554/eLife.66376 (PMC8806190; doi:10.7554/eLife.66376)
Supplement: Supplementary file 1. — (a) MprF-directed antibodies and its target peptides. (b) Bacterial strains used in this study. (c) Plasmids used in this study. (d) Primers used in this study. [file elife-66376-supp1.docx]

**Supplementary file 1**

***Supplementary file 1a.***

| **Antibody** | **Antigen** | **Peptide sequence** | **Framework** |
| --- | --- | --- | --- |
| M-C1 | Cyclic peptide, loop TMS 1-2 | ELSGINFKDTLVEFSKINR | VH3-23 kappa 3 |
| M-C7.1 | Cyclic peptide, loop TMS 7-8 | LGFKTLGVPEEKV | VH1A kappa 1 |
| M-C7.2 | Cyclic peptide, loop TMS 7-8 | LGFKTLGVPEEKV | VH3-23 kappa 1 |
| M-C7.3 | Cyclic peptide, loop TMS 7-8 | LGFKTLGVPEEKV | VH1A kappa 1 |
| M-C9.1 | Cyclic peptide, loop TMS 9-10 | DALYDGNHLT | VH1A kappa 1 |
| M-C9.2 | Cyclic peptide, loop TMS 9-10 | DALYDGNHLT | VH3-23 kappa 1 |
| M-C13.1 | Cyclic peptide, loop TMS 13-14 | DIYTIEMHTSVLR | VH1A kappa 1 |
| M-C13.2 | Cyclic peptide, loop TMS 13-14 | DIYTIEMHTSVLR | VH1A kappa 1 |

***Supplementary file 1b.***

| Strain name | Characteristics |
| --- | --- |
| *S. aureus* SA113 WT (ATCC 35556) | Restriction-deficient *S. aureus* strain derived from NCTC 8325 [1] |
| *S. aureus* SA113*∆mprF* | *mprF* deletion mutant of SA113, gene replaced by erythromycin resistance cassette [2]. |
| *S. aureus* SA113*∆spa* | *spa* deletion mutant of SA113. Constructed in this study. Markerless. |
| *S. aureus* SA113*∆spa∆mprF* | *spa* and *mprF* double deletion mutant of SA113. Constructed in this study. Erm^r^ |
| *S. aureus* DAP-R MRSA 703 | Daptomycin resistant clinical CA-MRSA isolate possessing a single point mutation in *mprF* (S295L) [3] |
| *S. aureus* USA300 LAC | CA-MRSA WT strain [4] |
| *E. coli* TG1 | Strain for phage display usage [5] |

***Supplementary file 1c.***

| **Plasmid** | **Characteristics** | **Short name in figures** |
| --- | --- | --- |
| pKOR1 | *E. coli/S. aureus* shuttle vector to allow allelic replacement with inducible counter-selection in staphylococci [6] | - |
| pRB474 | *E. coli/S. aureus* shuttle vector [7] | pRB |
| pRB474mprF | *mprF* cloned in *E. coli/S. aureus* shuttle vector pRB474 [2] | pRB-MprF |
| pRB474mprF-GFP | N-terminally GFP-tagged *mprF* cloned in *E. coli/S. aureus* shuttle vector pRB474 [8] | pRB-MprF-GFP |
| pRB474mprFdelCys flag | C-terminally FLAG®-tagged, cysteine codon-depleted *mprF* cloned in *E. coli/S. aureus* shuttle vector pRB474; constructed in this study | WT (-Cys) |
| pRB474mprFdelCys T263C flag | C-terminally FLAG®-tagged, cysteine codon-depleted *mprF* with artificial cysteine insertion cloned in *E. coli/S. aureus* shuttle vector pRB474; as indicated, each plasmid bears another amino acid substituted against cysteine (T263C, A99C, or T480C) | pRB-T263C |
| pRB474mprFdelCys A99C flag |  | pRB-A99C |
| pRB474mprFdelCys T480C flag |  | pRB-T480C |

***Supplementary file 1d.***

| **Primer** | **5’ 🡪 3’ sequence** | **Usage** |
| --- | --- | --- |
| A99C fw | GCATTGAATTGTATTGTAGGTTTCGGTGGCTTTATTGGTGCAGGCG | Forward primer for construction of pRB-A99C by site-directed mutagenesis |
| A99C rev | CCGAAACCTACAATACAATTCAATGCATTGATGATATAACTTACTC | Reverse primer for construction of pRB-A99C by site-directed mutagenesis |
| T263C fw | GTTGTATTACTAGGATTTAAATGTTTAGGTGTCCCTGAGGAAAAAG | Forward primer for construction of pRB-T263C by site-directed mutagenesis |
| T263C rev | CTTTTTCCTCAGGGACACCTAAACATTTAAATCCTAGTAATACAAC | Reverse primer for construction of pRB-T263C by site-directed mutagenesis |
| T480C fw | GGAACGTTATATGCATTAGATATTTATTGTATTGAAATGCATACATCTGTATTGCG | Forward primer for construction of pRB-T480C by site-directed mutagenesis |
| T480C rev | CGCAATACAGATGTATGCATTTCAATACAATAAATATCTAATGCATATAACGTTCC | Reverse primer for construction of pRB-T480C by site-directed mutagenesis |
| mprF C199+204S fw | TACTCTACTTTAGTGTCGTCTGTTGAATGGTTAGCAG | Primer for cysteine depletion of pRB474 encoded native *mprF* by site-directed mutagenesis |
| mprF C199+204S rev | AACAGACGACACTAAAGTAGAGTACAATCCTACAAAACG |  |
| mprF C217A fw | TTCGCTGGTGTAATTGTTGACGC |  |
| mprF C217A rev | ACCAGCGAAATATAATACAACTGC |  |
| mprF C380A fw | GCTGCTTTATTACTTTTACTGAATGTAGTTGG |  |
| mprF C380A rev | TAAAGCAGCACTAGTATGAATTGCC |  |
| mprF C526S fw | GATAGCGAGGAGATTATTAATCAG |  |
| mprF C526S rev | CTCGCTATCTTCAATTTTAGAAG |  |
| mprF C717S fw | GTAATTGCATTTAGTAGTTTAATGCCAACATACTTTAATGATG |  |
| mprF C717S rev | CTACTAAATGCAATTACTTCATTTTCTTCATTTCGCATTACACC |  |
| Spa-del_attB1 | ggggacaagtttgtacaaaaaagcaggccaatattccatggtccagaact | Construction of a markerless *spa* knockout mutant using the pKOR1 vector system [6] |
| Spa-del | gtcgagatctataaaaacaaacaatacacaacg |  |
| Spa-del_attB2 | ggggaccactttgtacaagaaagctgggatcagcaagaaaacacacttcc |  |
| Spa-del rev | aaaagatctaacgaattatgtattgcaata |  |
| MprF_USA300_fw | cagatatcaatatgacaaaag | Amplification of *mprF* in USA300 |
| MprF_USA300_rev | cttaaatattcttatctgtacc | Amplification of *mprF* in USA300 |
| MprF_USA300_600 | gtcatttttcttaccattattc | Sequencing of *mprF* in USA300 |
| MprF_USA300_1200 | gtgcttgtttattacttttac | Sequencing of *mprF* in USA300 |
| MprF_USA300_1800 | gttaggtgatgaaaatgcc | Sequencing of *mprF* in USA300 |
| MprF_USA300_2200 | gatggttgccagagttag | Sequencing of *mprF* in USA300 |
| MprF_USA300_800rev | cttcttagctgatgtacc | Sequencing of *mprF* in USA300 |

**Supplemental references**

1. Iordanescu, S. and M. Surdeanu, *Two restriction and modification systems in Staphylococcus aureus NCTC8325.* J Gen Microbiol, 1976. **96**(2): p. 277-81.

2. Peschel, A., et al., *Staphylococcus aureus resistance to human defensins and evasion of neutrophil killing via the novel virulence factor MprF is based on modification of membrane lipids with l-lysine.* J Exp Med, 2001. **193**(9): p. 1067-76.

3. Jones, T., et al., *Failures in clinical treatment of Staphylococcus aureus Infection with daptomycin are associated with alterations in surface charge, membrane phospholipid asymmetry, and drug binding.* Antimicrob Agents Chemother, 2008. **52**(1): p. 269-78.

4. Wang, R., et al., *Identification of novel cytolytic peptides as key virulence determinants for community-associated MRSA.* Nat Med, 2007. **13**(12): p. 1510-4.

5. Prassler, J., et al., *HuCAL PLATINUM, a synthetic Fab library optimized for sequence diversity and superior performance in mammalian expression systems.* J Mol Biol, 2011. **413**(1): p. 261-78.

6. Bae, T. and O. Schneewind, *Allelic replacement in Staphylococcus aureus with inducible counter-selection.* Plasmid, 2006. **55**(1): p. 58-63.

7. Bruckner, R., *A series of shuttle vectors for Bacillus subtilis and Escherichia coli.* Gene, 1992. **122**(1): p. 187-92.

8. Ernst, C.M., et al., *The lipid-modifying multiple peptide resistance factor is an oligomer consisting of distinct interacting synthase and flippase subunits.* MBio, 2015. **6**(1).
